# Comparison of selected metals in the fillers of 14 commercial hemp cigarette brands to commercial tobacco cigarettes

**DOI:** 10.1186/s42238-025-00330-7

**Published:** 2025-12-10

**Authors:** Naudia R. Gray, R. Steven Pappas, Clifford H. Watson

**Affiliations:** Tobacco & Volatiles Branch, Division of Laboratory Sciences, Centers for Disease Control and Prevention, 4770 Buford Hwy, MS S110-3, Atlanta, GA 30341 USA

**Keywords:** Hemp, Hemp cigarettes, Metals, Hemp filler, Inorganic, Cannabis, CBD, CBD cigarettes, Elements, NIST 8210

## Abstract

*Cannabis sativa* L. containing < 0.3% delta-9 tetrahydrocannabinol (THC) is currently defined as hemp. Many different legal products in the United States now contain hemp and are marketed for their cannabinoid effects, as an alternative to tobacco products, or even as an aid for tobacco smoking cessation. The hemp cigarettes analyzed have similar designs to tobacco cigarettes with a filter and filler wrapped in paper. *Cannabis sativa*, like tobacco (*Nicotiana tabacum*), is a hyperaccumulator of metals. Currently, no publications have reported analyses of metals in these cigarette-like products. Hemp and cannabidiol (CBD) products are increasing in popularity. Thus, reporting the metal concentrations from a variety of hemp cigarette brands can help assess the potential for harmful exposures. We analyzed the hemp filler in 14 commercial brands for beryllium (Be), chromium (Cr), manganese (Mn), cobalt (Co), nickel (Ni), arsenic (As), cadmium (Cd), lead (Pb), and uranium (U) content.

The hemp cigarette filler metals concentrations are compared to previously published metals levels in tobacco cigarette and little cigar filler. NIST Reference Material (RM) 8210 Hemp Plant was also analyzed to assess and confirm analytical accuracy. Of note, all hemp cigarette filler cadmium concentrations were below our lowest reportable level, and statistically lower than our previously published U.S. tobacco cigarettes and little cigars filler. The other metal concentration ranges were similar to previous tobacco cigarettes and little cigars results, although mean concentrations were statistically different in many cases. Different states have testing requirements with action limits for selected metals concentrations in *Cannabis sativa* L. Several hemp cigarette brands had chromium, nickel, arsenic, and lead concentrations that were above some state action limits.

## Background

*Cannabis sativa* refers to a genus of plant that includes both marijuana (> 0.3% delta-9 tetrahydrocannabinol (THC) by dry-weight) and hemp (< 0.3% THC by dry-weight) [1]. THC is considered the main psychoactive cannabinoid in the cannabis plant [1]. In the United States, marijuana is currently a Schedule I controlled substance under the Controlled Substance Act [1]. The Agricultural Improvement Act of 2018, also known as the Farm Bill, legalized industrial hemp in the United States [2]. Many hemp products, often sold for the presence of other cannabinoids, such as cannabidiol (CBD), are now available. Hemp products are increasing in popularity as they are advertised to help with relaxation, focus, pain relief, and improvement of well-being [3–9]. Hemp cigarettes, a relatively new product, are now sold by multiple companies in the United States. Hemp cigarettes are sometimes labeled or called CBD cigarettes. The content of the cannabinoid of interest, often CBD, is advertised per pack or per cigarette on websites and packaging. Hemp cigarette filler usually contains only hemp (flower and/or trim) but may also contain spices, such as peppermint [3], sage [4], other plants such as mullein [4], or food grade flavoring [3]. The filler is wrapped in paper (plant fiber, hemp, wood pulp, etc.) with a filter, somewhat visually similar to tobacco cigarettes. Some hemp cigarettes brands label their products as an alternative to tobacco smoking because they are free from tobacco, nicotine, chemicals, and additives [5–7, 10]. These claims have not been evaluated or approved by the Food and Drug Administration.

Although hemp and tobacco cigarettes share similar designs, peer-reviewed publications on the analyses of hemp cigarettes are very limited. Literature searches using Google Scholar and PubMed for keywords, such as “metals,” “elements,” “hemp cigarettes,” and “CBD cigarettes” returned a limited number of publications, none related to the analysis of metals in hemp cigarette filler or smoke. Ward et al. reported carbonyls in mainstream smoke from two different hemp cigarettes [11].

*Cannabis sativa* L., as well as tobacco (*Nicotiana tabacum*), are tolerant to the accumulation of metals. However, metal concentrations in both plants largely depend on soil metals concentrations and soil conditions where the plant was grown [12, 13]. There have been limited peer-reviewed publications on metals in the industrial United States hemp flower itself. In 2024, Wright et al. published data on 26 elements from CBD cultivars [14]. Various vendor application notes also provide data on limited hemp plant samples [15–17].

All the hemp cigarette companies in the current study provide lab reports as quality assurance checks with cannabinoid results that also verify the hemp used in the cigarettes have < 0.3% THC [10, 18–28]. Of note, Wild Hemp Hempettes^®^ Virginia Style report was not found on the company website, although the other flavors of Hempettes^®^ provided separate lab reports. Some lab reports contain concentration results for a limited set of metals (arsenic, cadmium, lead, and mercury) and other analytes of concern. The metals concentrations are compared to state action limits to pass or fail testing requirements. A few states require testing of additional metals, such as chromium and nickel. However, action limits for the metals are not consistent between states.

This study applies our validated method for metals in the tobacco plant [29, 30] to hemp filler as a non-tobacco plant material used as a combustible product. The selected metals for analysis were beryllium (Be), chromium (Cr), manganese (Mn), cobalt (Co), nickel (Ni), arsenic (As, metalloid), cadmium (Cd), lead (Pb), and uranium (U). The selection criteria [29, 30] for metals analyzed in this study included available toxicity reports and their inclusion in the Food and Drug Administration’s Harmful and Potentially Harmful Constituents (HPHC) in Tobacco Products and Tobacco Smoke list [31]. The HPHC list categorizes our analytes as carcinogens (Be, Cr, Co, Ni, As, Cd, Pb, U), respiratory toxicants (Cr, Ni, Cd, U), cardiovascular toxicants (Co, As, Pb), and reproductive or developmental toxicants (Cr, As, Cd, Pb) [31]. Manganese is not included in the HPHC list. However, it can cause neurotoxicity and sensitization, as well as pulmonary inflammation through oxidation-reduction processes [32–34]. Because of the similar designs between tobacco and hemp cigarettes, our analyses included examining 14 brands of hemp cigarettes in triplicate for metals concentrations in filler along with NIST RM 8210 hemp reference material [35]. Brands were chosen based on market availability. NIST RM 8210 is a hemp reference material that provides non-certified metal concentrations. We analyzed NIST RM 8210 to further demonstrate our method’s accuracy and precision in hemp material.

To help put these findings into perspective, we compare metal concentrations of filler from hemp cigarettes to those of little cigars [30] and tobacco cigarettes [29]. As a similar combustible plant product, these hemp cigarettes can serve as a tobacco and nicotine-free matrix with no further method modifications required to analyze. We also compared our metals concentrations to those provided by the vendors (third-party laboratory results) and to established state limits. This report provides important information on the potential hyperaccumulation of metals in the hemp plant by reporting the magnitude of selected metals concentrations in hemp cigarettes filler.

## Materials & methods

14 different commercially available hemp cigarette brands were obtained through Lab Depot (Dawsonville, GA, USA) and assigned unique pack IDs. Packs were stored in original packaging at -20 °C until analysis.

### Metals in hemp cigarette filler & NIST 8210 hemp plant

The filler from three hemp cigarettes per pack was used for each replicate. Filler was prepared and analyzed, with noted exceptions, according to a previously published method validated under our ISO 17025 laboratory scope of accreditation [30]. The combined filler from the three cigarettes was dried at 100 °C in an oven (Binder, Inc.; Bohemia, NY, USA) for 16 h. Filler was ground using a coffee grinder (Mr. Coffee; Cleveland, OH, USA) to increase homogeneity as previously described [11].

Dried filler (0.100–0.150 g) was digested in MARS Xpress modified polytetrafluoroethylene (TFM) digestion vessels with a CEM MARS 6 microwave system (Matthews, NC, USA) using 9 mL distilled nitric acid (Environmental grade, GFS Chemicals, Powell, OH, USA, further purified with a Savillex perfluoroalkoxy resin polymer (PFA) sub-boiling still, Minnetonka, MN, USA), 0.5 mL Veritas^®^ double distilled hydrofluoric acid (GFS Chemicals), and 0.5 mL stabilizer free hydrogen peroxide (30% (w/w) for ultratrace analysis, Sigma-Aldrich; St. Louis, MO, USA). The Plant Material OneTouch™ program was used with a 20–25 min ramp to 200 °C and 10 min hold at 200 °C. A procedural blank was prepared by adding nitric acid, hydrofluoric acid, and hydrogen peroxide and digesting and preparing along with samples. The digest was diluted to 100 mL in a class A flask using ultrapure water (AquaSolutions; Jasper, GA, USA).

Diluted samples were analyzed for beryllium, chromium, manganese, cobalt, nickel, arsenic, cadmium, lead, and uranium using an Agilent 8800 QQQ-ICP-MS (triple quadrupole-inductively coupled plasma-mass spectrometer; Santa Clara, CA, USA). Selenium was included in the previous method [30] but was eliminated from the current method due to low concentrations in tobacco and low health risk. Five calibration standards were prepared in 9% nitric acid and 0.5% hydrofluoric acid using dilutions of High Purity Standards (Charleston, SC, USA) for all analytes. Instrument modes and internal standard assignments were as previously described [30] with exceptions. Manganese was analyzed in MS-MS mode with helium cell gas and kinetic energy discrimination and ^103^Rh+ internal standard in the updated method, with ^55^Mn^+^ as the quantitated mass. In the current method, lead is quantitated as the sum of ^206, 207, 208^Pb^+^. Internal standard solution was prepared as previously published [30] and was diluted at a 1:1 ratio with the digested samples using a tee. Slight adjustments to instrument settings and setup since the last publication include 0.38 mm internal diameter peristaltic pump tubing to introduce the sample and internal standards. Cell gas flows in the respective modes were 5 mL/min (helium mode), 0.7 mL/min (oxygen mode), and 3.5 mL/min (ammonia mode) as 10% ammonia/90% helium.

Procedural digestion blank was blank subtracted from samples. Quality control (QC) tobacco samples 1S3 (reference tobacco; North Carolina State University, Raleigh, NC, USA) and INCT-PVTL-6 (Polish Virginia Tobacco Leaves certified reference material; Instytut Chemii I Techniki Jądrowej; Warszawa, Poland) were prepared using the same procedure as samples and analyzed in duplicate, bracketing the samples. Quality control evaluation was as previously published [30]. The use of reference materials as quality control samples provides homogenous tobacco material to demonstrate the precision of the analytical method. Although the shelf life of INCT-PVTL-6 was established until the end of 2020 [36], samples analyzed after the established shelf life meet our characterization criteria to be used as a quality control sample.

Method Limits of Detection (LODs) were calculated with the equation. [37] and verified:

LOD= [mean_procedural blank_ + 1.645 + (S_procedural blank_ + B)] / (1–1.645 X A).

Mean_procedural blank_ and S_procedural blank_ (standard deviation) were calculated using the data from 20 procedural digest blanks. Factors A (slope) and B (intercept) were calculated using Taylor’s method [38] of plotting mean and standard deviations versus mean concentrations using digests of procedural blanks, 1S3, INCT-PVTL-6, CTA-OTL-1 (Oriental Tobacco Leaves certified reference material; Instytut Chemii I Techniki Jądrowej; Warszawa, Poland), NIST SRM (Standard Reference Material) 1570a (spinach leaves), and NIST SRM 1573a (tomato leaves) over 20 analytical runs.

To determine dry-mass per cigarette and moisture content, the filler from one cigarette per pack (*n* = 3) was weighed before and after drying at 100 °C for 16 h.

## Results

NIST RM 8210 was analyzed as-is and converted to dry-mass using the given 5.46% moisture content calculated by NIST (0.9454 correction factor) [35]. Table 1 displays results (average ± standard deviation) compared to the non-certified values (average ± expanded uncertainty (95%)) of the reference material on a dry-mass basis [35]. Four analytes are below our reportable levels since our procedures’ manual prohibits reporting concentrations outside analytical calibration ranges. All our results were within NIST’s reported expanded uncertainties except for the manganese average (159 µg/g) that was slightly above NIST’s uncertainty range (117.6–157.6 µg/g; analyzed by ICP-OES (inductively coupled plasma-optical emission spectroscopy)). Our reported manganese average still gives an 84% accuracy compared with NIST’s uncertified value [35].

**Table 1:** Average ± standard deviation (*n* = 3) of NIST RM 8210 hemp (analyzed as-is and converted to dry mass) with comparison to NIST certificate of analysis (analyzed on dry-mass basis) non-certified values [35] (average ± expanded uncertainty (95%)) (µg/g)

	Be	Cr	Mn	Co	Ni	As	Cd	Pb	U
**CDC Dry-Mass**,** corrected**^ǂ^	< LSTD (0.0050)	0.587 ± 0.055	159 ± 3	0.204 ± 0.012	3.94 ± 0.11	< LSTD (0.050)	< LSTD (0.500)	0.240 ± 0.089**	< LSTD (0.010)
**NIST Non-Certified Dry-Mass** ^#^	0.0023 ± 0.0019	0.552 ± 0.170	137.6 ± 20.0	0.196 ± 0.035	3.98 ± 0.73	0.043 ± 0.012	0.083 ± 0.014	0.211 ± 0.060	0.0044 ± 0.0013

beryllium (Be), chromium (Cr), manganese (Mn), cobalt (Co), nickel (Ni), arsenic (As), cadmium (Cd), lead (Pb), uranium (U); LSTD (lowest standard)

**2 replicates were < LSTD

^ǂ^Average ± Standard Deviation; ^#^ Average ± Expanded Uncertainty (95%)

The average filler metal (beryllium, chromium, manganese, cobalt, nickel, arsenic, cadmium, lead, and uranium) concentrations (*n* = 3) are summarized in Table 2. They are reported on a dry-mass basis as µg/g. Limits of detection and lowest standards are also included in Table 2.

For the 14 hemp cigarettes brands, cadmium was below lowest standard (< LSTD, 0.500 µg/g) in every sample, although > the method LOD (0.016 µg/g). Therefore, comparisons of cadmium concentrations between hemp cigarettes brands are not reported. Beryllium (< LSTD to 0.0895 µg/g) and uranium (< LSTD to 0.283 µg/g) were among the lowest reportable concentrations. However, there were some equally low cobalt (0.165 to 0.726 µg/g) and arsenic concentrations (0.0527 to 0.638 µg/g). Ranges for chromium concentrations were 0.566 to 3.14 µg/g; nickel concentrations were 1.68 to 5.47 µg/g; and lead concentrations were < LSTD to 1.06 µg/g. Manganese concentrations were highest, ranging from 124 to 355 µg/g. Lucky Leaf had the lowest metals concentrations for beryllium, chromium, cobalt, arsenic, and uranium compared to the other brands. Chief Stix™ had the lowest manganese concentration; Oklahoma Smokes Menthol had the lowest nickel concentration; and Chief Stix™ and Neurogan^®^ had the lowest lead concentrations. CBD American Shaman™ Shaman Smokes Original had the most reportable analytes (chromium, manganese, cobalt, arsenic, and lead) with the highest results compared to the other brands. Oklahoma Smokes Original was the highest for beryllium and uranium, and Mountain^®^ Pineapple Squeeze was the highest for nickel.

Dry mass of filler per cigarette (g/cig), *n* = 3, is shown in Table 2 with ranges from 0.537 ± 0.039 g/cig for Neurogan^®^ to 0.847 ± 0.011 g/cig for CBD American Shaman™ Shaman Smokes Original. Some hemp cigarettes brands had a noticeably wider range of dry mass of filler in g/cig than others. For example, the %RSD (relative standard deviation) of dry mass/cig was 1% RSD for CBD American Shaman™ Shaman Smokes Original and up to 13% RSD for Plain Jane™ Menthol.

**Table 2:** Hemp cigarettes filler average concentration for selected metals (average ± standard deviation, µg/g, *n* = 3)

	Be	Cr	Mn	Co	Ni	As	Cd	Pb	U	Dry Weight per Cigarette (g/cig)	
LOD	0.0027	0.089	0.056	0.013	0.110	0.031	0.016	0.012	0.0022		
LSTD assuming 100 mg sample	0.0050	0.200	20.0	0.100	0.500	0.050	0.500	0.200	0.010	Ave. ± Std. Dev.	%RSD
**Plain Jane™ Menthol**	0.0175 ± 0.0013	1.55 ± 0.66	211 ± 9	0.262 ± 0.014	3.78 ± 0.24	0.0799 ± 0.0061	< LSTD	0.214 ± 0.006	0.0161 ± 0.0007	0.841 ± 0.111	13%
**Mountain** ^®^ **Pineapple Squeeze**	0.0310 ± 0.0073	1.64 ± 0.23	163 ± 11	0.431 ± 0.043	5.47 ± 0.38	0.115 ± 0.038	< LSTD	0.297 ± 0.050	0.0289 ± 0.0062	0.741 ± 0.019	3%
**Crème Peppermint by Sugar™**	0.0112 ± 0.0012	1.11 ± 0.08	170 ± 10	0.273 ± 0.018	2.65 ± 0.13	0.124 ± 0.013	< LSTD	0.419 ± 0.042	0.0145 ± 0.0003	0.782 ± 0.012	2%
**Hemp Rolls** ^®^	0.00885 ± 0.00403*	1.53 ± 0.65	190 ± 12	0.250 ± 0.035	4.52 ± 0.05	0.154 ± 0.019	< LSTD	0.370 ± 0.059	0.0164 ± 0.0094	0.822 ± 0.095	12%
**CBD American Shaman™ Shaman Smokes Original**	0.0753 ± 0.0182	3.14 ± 0.23	355 ± 70	0.726 ± 0.050	3.23 ± 0.24	0.638 ± 0.334	< LSTD	1.06 ± 0.30	0.258 ± 0.075	0.847 ± 0.011	1%
**BLĀZ**	0.0693 ± 0.0016	2.89 ± 0.13	153 ± 9	0.606 ± 0.022	3.34 ± 0.51	0.257 ± 0.016	< LSTD	1.03 ± 0.13	0.118 ± 0.009	0.640 ± 0.067	11%
**Redwood Reserves**	0.0211 ± 0.0025	1.06 ± 0.27	235 ± 26	0.230 ± 0.006	3.20 ± 0.23	0.0579 ± 0.0072	< LSTD	0.209 ± 0.017	0.0122 ± 0.0014	0.716 ± 0.059	8%
**Oklahoma Smokes Original**	0.0895 ± 0.0057	2.95 ± 0.86	137 ± 8	0.418 ± 0.003	1.73 ± 0.06	0.370 ± 0.024	< LSTD	0.982 ± 0.042	0.283 ± 0.013	0.622 ± 0.026	4%
**Oklahoma Smokes Menthol**	0.0712 ± 0.0117	1.89 ± 0.18	132 ± 6	0.368 ± 0.033	1.68 ± 0.10	0.329 ± 0.032	< LSTD	0.797 ± 0.089	0.216 ± 0.016	0.587 ± 0.011	2%
**Wild Hemp Hempettes** ^®^ **Original**	0.0525 ± 0.0034	2.54 ± 0.50	237 ± 16	0.496 ± 0.072	3.09 ± 0.39	0.165 ± 0.002	< LSTD	0.593 ± 0.027	0.0763 ± 0.0072	0.617 ± 0.023	4%
**Wild Hemp Hempettes** ^®^ **Virginia Style**	0.0449 ± 0.0034	1.96 ± 0.15	210 ± 7	0.450 ± 0.020	2.88 ± 0.018	0.138 ± 0.016	< LSTD	0.451 ± 0.063	0.0578 ± 0.0033	0.792 ± 0.015	2%
**Lucky Leaf**	< LSTD	0.566 ± 0.132	151 ± 4	0.165 ± 0.007	2.92 ± 0.16	0.0527 ± 0.0039	< LSTD	0.201 ± 0.029*	< LSTD	0.764 ± 0.053	7%
**Chief Stix™**	0.0107 ± 0.0046	1.08 ± 0.30	124 ± 31	0.195 ± 0.029	2.96 ± 0.32	0.0799 ± 0.0323	< LSTD	< LSTD	0.0111 ± 0.0013*	0.623 ± 0.060	10%
**Neurogan** ^®^	0.00933 ± 0.00105	1.24 ± 0.28	222 ± 21	0.265 ± 0.036	5.20 ± 0.34	0.112 ± 0.013	< LSTD	< LSTD	0.0104 ± 0.0003	0.537 ± 0.039	7%

*One replicate was < LSTD

beryllium (Be), chromium (Cr), manganese (Mn), cobalt (Co), nickel (Ni), arsenic (As), cadmium (Cd), lead (Pb), uranium (U); LOD (limit of detection); LSTD (lowest standard); RSD (relative standard deviation)

## Discussion

### NIST RM 8210 results

Although NIST provides only non-certified values for our analytes in RM 8210, we can compare our results to the non-certified values to aid in determination of suitability of the method for use with a hemp matrix. Of note, we prepared the finely ground NIST RM 8210 hemp as-is using 0.100–0.150 g of sample according to our method, as opposed to the 0.5 g that is instructed in the NIST certificate [35]. The 5.46% moisture determined by NIST was used for our moisture corrections. Results were corrected from “as-is” to dry-mass results according to RM 8210 instructions and compared to the certificate of analysis.

All results were within the expanded standard uncertainty of the non-certified values determined at NIST using ICP-OES except for manganese. Since the NIST values are non-certified, we nevertheless reported our results here based on our long-term method quality control measurements. Overall, our NIST RM 8210 results presented confidence that our method (originally developed for tobacco matrix) can be applied to hemp. This was further verified in several proficiency testing schema, including NIST CannaQAP hemp plant samples [39], the ASTM PTP (proficiency testing program) hemp flower program, and Food Analysis Performance Assessment Scheme (FAPAS® ) proficiency testing schema (including chili powder, infant cereal, and soy flour). In the 3 ASTM PTP test cycles of 2024 for metals analysis, our results gave z-scores within − 1 to 1 for arsenic and lead. Our cadmium results were < lowest reportable level (LRL) and the other analytes did not have statistics performed in the report. This interspecies applicability was also demonstrated in analyses of NIST tomato and spinach leaves plant SRMs [30].

### Comparison to tobacco cigarettes

We previously reported the concentrations of selected metals in fillers of little cigars and tobacco cigarettes from the United States [29, 30]. Caruso et al. also reported chromium, nickel, arsenic, cadmium, and lead in U.S. tobacco cigarettes filler by polarized energy dispersive x-ray fluorescence (XRF) [31]. In general, Caruso’s mean results were similar but slightly lower than our published results for the same analytes [29, 40].

**Fig. 1:**
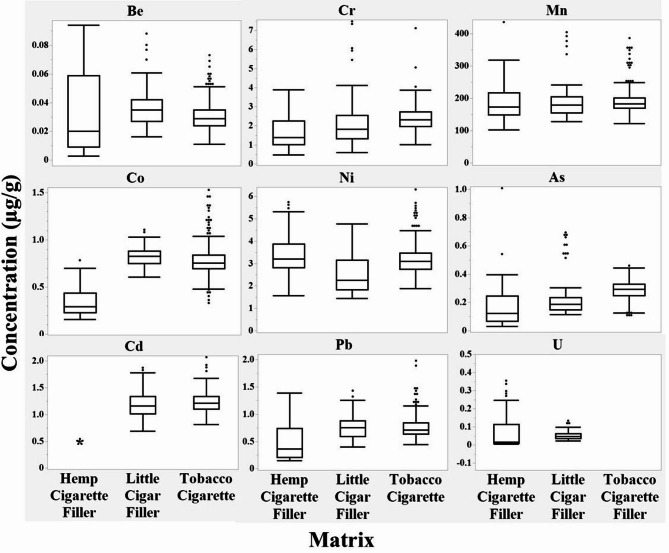
Box and whisker plot of beryllium (Be), chromium (Cr), manganese (Mn), cobalt (Co), nickel (Ni), arsenic (As), cadmium (Cd), lead (Pb), and uranium (U) for 14 hemp cigarette brands (*n* = 3), 17 little cigar brands (*n* = 5) [30], and 50 tobacco cigarette brands (*n* = 7) [29] commercially sold in the United States. The data from the 50 tobacco cigarette brands did not include uranium, therefore, only hemp cigarettes and little cigars data are shown in the uranium plot. *All hemp cigarette filler cadmium results were < LSTD (0.500)

Hemp cigarettes filler results from Table 2 were compared to 17 brands of U.S. little cigars filler, a variation on the design of tobacco cigarettes [30]. Results from 50 brands of U.S. tobacco cigarettes filler [29], which included all analytes in this panel except for uranium, were also compared. Box and whisker plots for each analyte using JMP^®^ 17.0.0 (JMP Statistical Discovery, LLC; Cary, NC) are shown in Fig. 1. The box represents the interquartile range (25th to 75th percentile). The line inside the box represents the median. Whiskers are the minimum and maximum data points within 1.5 times the interquartile range. Points outside are outliers. Table 3 shows the overall mean ± standard deviation from all brands and replicates from the hemp cigarettes, little cigars [30], and tobacco cigarettes [29] filler studies. JMP^®^ 17.0.0 was used to perform Tukey-Kramer HSD (honestly significant difference) to determine if there were statistical mean differences (*p* < 0.05) between hemp cigarettes and the other matrices (Table 3).

**Table 3:** Overall mean concentrations ± standard deviations of metals in filler from hemp cigarettes, little cigars [30], and tobacco cigarettes [29] with p-values to compare means of hemp cigarettes to little cigars and tobacco cigarettes

	Be	Cr	Mn	Co	Ni	As	Cd	Pb	U
**Hemp Cigarettes**	0.0340 ± 0.0296	1.70 ± 0.88	189 ± 61	0.352 ± 0.163	3.37 ± 1.06	0.175 ± 0.171	< LSTD	0.474 ± 0.330	0.0796 ± 0.104
**Little Cigars**	0.0367 ± 0.0132	2.13 ± 1.31	187 ± 54	0.814 ± 0.106	2.56 ± 0.90	0.234 ± 0.146	1.19 ± 0.30	0.755 ± 0.202	0.0518 ± 0.0225
	*p* = 0.5166	*p* = 0.0111	*p* = 0.9903	*p* < 0.0001	*p* < 0.0001	*p* = 0.0031	*p* < 0.0001	*p* < 0.0001	*p* = 0.0186
**Tobacco Cigarettes**	0.0302 ± 0.0095	2.36 ± 0.63	190 ± 37	0.780 ± 0.157	3.16 ± 0.65	0.287 ± 0.065	1.23 ± 0.19	0.760 ± 0.195	n/a
	*p* = 0.1673	*p* < 0.0001	*p* = 0.9881	*p* < 0.0001	*p* = 0.1734	*p* < 0.0001	*p* < 0.0001	*p* < 0.0001	n/a

Tukey-Kramer HSD (honestly significant difference) was used to determine statistically significant differences (*p* < 0.05)

Figure 1 shows that most analyte concentrations for hemp cigarette filler overlap that from little cigar filler and tobacco cigarette filler of the same analyte. However, using JMP^®^ 17.0.0 to perform the Tukey-Kramer HSD (honestly significant difference), most analytes had significant mean differences (*p* < 0.05) between hemp cigarettes filler and little cigars and tobacco cigarettes filler. Beryllium has a larger concentration range in hemp cigarettes filler than in little cigars and tobacco cigarettes fillers. Nevertheless, the hemp cigarettes beryllium mean was not statistically different from either little cigars or tobacco cigarettes. Uranium also has a larger concentration range in hemp cigarettes than little cigars (uranium was not included in the tobacco cigarettes analysis). In this case, the hemp cigarettes mean was statistically higher than little cigars. The chromium concentrations in hemp cigarettes were similar to those found in traditional tobacco cigarettes. The Lucky Leaf brand had the only average chromium concentration (0.566 ± 0.132 µg/g) lower than previous tobacco cigarettes chromium concentrations. The Lucky Leaf description states that indoor and outdoor trim were used [6]. The use of indoor grown plants could affect metal concentrations by allowing for greater control over the growing conditions. The use of trim, as opposed to the whole flower, could also affect metal concentrations although the hemp variety used may influence metal uptake to a greater extent [41]. There were no hemp cigarettes results with chromium higher than previously reported in little cigars or tobacco cigarettes filler. Despite these similarities, chromium hemp cigarettes mean was statistically lower than both little cigars and tobacco cigarettes fillers. Manganese concentrations in hemp cigarettes were similar to little cigars and tobacco cigarettes manganese concentrations with only Chief Stix™ (124 ± 31 µg/g) below the published tobacco ranges. Hemp cigarettes manganese mean was not statistically different from either little cigars or tobacco cigarettes fillers. Most hemp cigarettes concentrations for cobalt, arsenic, and lead are lower than the respective little cigars and tobacco cigarettes filler concentration ranges, and hemp cigarettes averages for these analytes were all significantly lower than in both tobacco fillers. Hemp filler nickel concentrations were within little cigars and tobacco cigarettes fillers ranges except for higher results for Mountain^®^ Pineapple Squeeze (5.47 ± 0.38 µg/g), Hemp Rolls^®^ (4.52 ± 0.05 µg/g), and Neurogan^®^ (5.20 ± 0.34 µg/g). While hemp cigarettes nickel mean concentration was not significantly different than tobacco cigarettes, it was significantly higher than little cigars.

Cadmium had the most noteworthy significant difference in metal concentrations in hemp filler (< LSTD) compared to little cigars and tobacco cigarettes filler (all reportable). This may be due to differences in cadmium uptake in *Cannabis sativa* L. compared to *Nicotiana tabacum*. A 2024 study by Guo et al. studied 9 hemp plant varieties grown in soils heavily contaminated with arsenic, copper, cadmium, and lead [41]. They found that most of the 4 elements were retained in the roots, and cadmium had the poorest uptake from the soil to the plant. Nash et al.’s 2024 study grew *Cannabis sativa* L. plants hydroponically with 10 mg/L cadmium exposure compared to control plants [42]. They also found that cadmium accumulation in the plant occurred mostly in the roots with 1448 mg/kg cadmium found in the root and 23.2 mg/kg cadmium found in the leaves after harvesting. In contrast, *Nicotiana tabacum* plants have the highest cadmium uptake to the leaves with lower accumulation in the roots and stems [43, 44].

### Comparison to published hemp data

The hemp filler metal concentrations reported here may also be compared to published data on cannabis plant samples. Data from these studies are shown in Table 4 for our 9 analytes of interest. Application notes from analytical instrumentation and equipment manufacturers provide small sample size reports on metals concentrations in hemp [15–17]. In Wright et al.’s study, 18 hemp samples marketed for smoking were purchased from retailers and 73 hemp samples were obtained from Michigan in a United States Department of Agriculture (USDA) study [14]. Coffman and Gentner analyzed cannabis leaves from plants grown in a greenhouse using 11 different Maryland soils [45]. Some studies outside of the United States were also included in Table 4. Zafeiraki et al. analyzed leaves/flowers of 90 hemp samples from Greece [46]. The Douvris et al. study analyzed 12 cannabis samples of leaves/flowers from illegal farms and 22 cannabis samples seized by law enforcement in Ghana [47]. Kuras and Wachowicz analyzed 20 hemp fiber samples from soil grown without fertilizer and 65 cannabis samples seized from law enforcement in Poland [48]. Shibuya et al. analyzed 153 cannabis samples seized by Brazilian law enforcement [49]. Landi’s results shown in Table 4 are from the analysis of inflorescences (flower clusters) of 6 different cannabis populations from growing 4 commercial cultivars of cannabis in varying conditions in Italy [50]. Nava et al. analyzed inflorescences from 6 varieties of hemp grown in 4 regions in Italy sampled in triplicate [51]. Khan et al.’s study collected 5 composite samples of cannabis plants that were growing in 2 different locations in Pakistan, and the leaves were dried, ground, and analyzed [52]. Kumar et al. analyzed cannabis samples purchased at a local market in India after drying and grinding [53]. Zerihuan et al. collected samples from 4 regions of Ethiopia and dried and ground the leaves before analysis [54]. Ćaćić et al. analyzed dried and ground leaves from 4 varieties of hemp grown in an open greenhouse using acidic and alkaline soils with no further treatment [55].

**Table 4:** Summary of cannabis results from other published studies (µg/g)

	Be	Cr	Mn	Co	Ni	As	Cd	Pb	U
**PerkinElmer (U.S.) **[15]	n/a	n/a	n/a	n/a	n/a	0.027–0.045†	0.029–0.042†	0.009–0.021†	n/a
**Agilent (U.S.) **[17]	0.0037–0.0053	0.081–0.273	114–230	0.143–0.162	0.108–0.186	0.026–0.160	0.0075–0.011	0.024–0.055	0.0032–0.0048
**CEM (U.S.) **[16]	n/a	n/a	n/a	n/a	n/a	0.0231 ± 0.0014	ND	0.163 ± 0.0018	n/a
**Wright (U.S.-retail) **[14]*	< 0.01 (0.014)	0.1 (0.7)	126 (318)	0.061 (0.271)	0.44 (1.75)	0.03 (0.41)	0.112 (1.329)	0.026 (2.105)	0.002 (0.019)
**Wright (U.S.-USDA) **[14]*	0.0017 (0.0045)	0.2 (0.9)	142 (247)	0.038 (0.068)	0.27 (0.66)	0.03 (0.10)	0.060 (0.527)	0.083 (0.181)	0.003 (0.005)
**Coffman (U.S.) **[45]	n/a	n/a	76–602	n/a	n/a	n/a	n/a	n/a	n/a
**Zafeiraki (Greece) **[46]	n/a	0.337–7.89	76.9–519	0.070–1.61	1.76–49.0	0.031–0.742	0.0070–0.431	0.095–1.75	0.0020–0.365
**Douvris (Ghana) **[47]	n/a	n/a	73–2,363	n/a	n/a	ND – 0.242	ND – 0.181	0.011–0.854	n/a
**Kuras (Poland) **[48]	n/a	n/a	34–493	n/a	n/a	n/a	n/a	0.20–3.40 (16.77^‡^)	n/a
**Shibuya (Brazil) **[49]	n/a	n/a	69–2,066	< 0.02–2.5	n/a	n/a	n/a	0.1–9.5	< 0.02–0.36
**Landi (Italy) **[50]	n/a	n/a	23–78	n/a	n/a	n/a	n/a	n/a	n/a
**Nava (Italy) **[51]	n/a	< LOD – 0.47	1.67–72.21	< LOD – 0.93	0.01–2.11	< LOD – 0.01	< LOD – 0.06	< LOD – 0.79	n/a
**Khan (Pakistan) **[52]	n/a	4.65–4.18	n/a	n/a	3.26–3.56	n/a	0.033–0.066	6.09–6.66	n/a
**Kumar (India) **[53]	n/a	48.41 ± 6.31	n/a	n/a	n/a	n/a	0.23 ± 0.08	31.17 ± 0.30	n/a
**Zerihuan (Ethiopia) **[54]	n/a	3.6–7.6	n/a	n/a	124–172	n/a	3.2–4.7	7.9–10.2	n/a
**Ćaćić (Croatia) **[55]	n/a	1.59–4.35	n/a	0.05–0.51	0.13–7.33	0.25–0.25	0.03–0.31	0.36–4.86	n/a

beryllium (Be), chromium (Cr), manganese (Mn), cobalt (Co), nickel (Ni), arsenic (As), cadmium (Cd), lead (Pb), uranium (U); *data presented as median (max); † results were below the study’s lowest standard; ‡ one sample only; ND = non-detect; n/a = not applicable

Some similar patterns are observed among our hemp cigarettes filler data shown in Table 2 and data shown in Table 4. Beryllium and uranium had the lowest results, and manganese had the highest results compared to other analytes. Most metals concentrations among those published in application notes [15–17], Wright et al. [14], Douvris (except for the large range of manganese) [47], and Landi [50] were lower than our hemp cigarettes filler results. Compared to our hemp cigarettes results, the majority of the metal concentrations of the other studies with larger sample sizes [45, 46, 48, 49] had larger ranges, with our results falling within those ranges.

There are several limitations to direct comparisons of Table 4 study results with our hemp cigarette filler results. Sample preparation and analysis techniques varied among the different studies. If proper techniques were not used to avoid contamination of metals during preparation or interferences were not accounted for during analysis, some studies could be reporting inaccurate metals data. For example, Zerihuan et al. mention glassware which should not be used for metal analysis because glass leaches metals and increases background [54]. To the best of our knowledge, none of the samples that were analyzed in Table 4 were from hemp cigarettes. Therefore, they did not go through the production process into a cigarette. Many studies had samples seized by law enforcement but did not go into further detail about the samples. While other study samples were from leaves or a combination of leaves and flowers from plants that were grown for research purposes. The parts of the plant analyzed can influence metal concentrations in the samples. Many of the samples were from plants grown under various soil conditions that may not accurately represent the conditions that would be used by hemp farmers. For example, plants with the highest manganese in the Coffman and Gentner study showed leaf chlorosis and stunted growth [45], and some samples in the Kuras and Wachowicz [48] study were grown without fertilizer. Location, which can influence soil and growing conditions, also differ among Table 4 studies. The cigarettes in our study were purchased from companies based across the United States with many company websites listing neither the geographic locations of hemp cultivation nor the conditions under which the hemp was grown. Various hemp cultivars analyzed in Table 4 studies and in this study may affect differences in metal concentrations. Guo et al. studied 9 hemp varieties for copper, arsenic, cadmium, and lead uptake from strongly contaminated soils [41]. They found that hemp variety and metal type were the main factors in growth and metal uptake [41].

### Comparison to state action limits

Some states have their own requirements for hemp/cannabis product testing including selected metals. Each state with testing requirements has its own regulations for manufacturers to follow if a product fails. For example, in Connecticut if a hemp product exceeds a metal limit, the manufacturer must dispose of the entire batch [56]. The metals and concentrations required for testing vary by state. These limits are often based on the United States Pharmacopeia’s (USP) permitted daily exposure (PDE) limits which are the maximum acceptable intake of the metal in a drug per day [57]. Limits can also be based on food and beverage, supplement, environmental, and cosmetic industry limits [57]. Adaptations of these established limits to cannabis can result in the inconsistencies among states. States may assume different daily dosages of cannabis which can vary depending on the user and may take into account the different routes of exposure which influence a metal’s toxicological impact [57].

Most commonly, states require cannabis product testing of arsenic, cadmium, mercury, and lead. Chromium is also required by New York [58], Michigan [59], Maryland [60], and Missouri [61]; and nickel is required by New York and Michigan. All except one of the 14 hemp cigarettes chromium concentrations were above the lowest 0.6 µg/g limit set by Maryland [60] and Missouri [61]. All nickel concentrations were above the lowest 1.0 µg/g limit set by Michigan [59]. California [62] has a limit of 0.2 µg/g arsenic and a 0.5 µg/g limit for lead. These concentrations are common to other states that have state product testing requirements. Four of the fourteen hemp cigarettes had arsenic values above 0.2 µg/g limit. Five of the fourteen hemp cigarettes had lead values above the 0.5 µg/g limit. Hemp cadmium concentrations were at or below our lowest reportable level, therefore, we could not compare hemp cadmium concentrations to any reporting limits. Increased testing for metals by hemp cigarette companies could reduce users’ exposures.

Of note, many of the tobacco cigarettes and little cigars would also be above the lowest chromium, nickel, arsenic, cadmium, and lead limits set for cannabis [29, 30]. All of the cadmium averages from tobacco product cigarettes were above the highest action limit for cannabis (0.5 µg/g) set by Colorado [29, 30, 63].

### Comparison to company-provided lab reports

Company-provided third-party lab reports were provided for the hemp cigarettes [18–28, 64]. However, a Wild Hemp Hempettes^®^ Virginia Style lab report was not available. Many laboratory reports also did not include information on a documented procedure to verify that the hemp batch tested was the same as the hemp used in a specific pack of hemp cigarettes. The data presented in the Chief Stix™ report is from a sample that the third-party lab analyzed after we received our hemp cigarette products [27]. The product description in the report matches our products, therefore, we compare below. Only 4 of the 14 reports included metals results. The reports are from third-party labs and hemp cigarette companies located in both Oregon and California. They compare concentrations to a pass/fail action limit that is set for hemp filler. In this case, all 4 reports were comparing against limits of 0.2 µg/g for arsenic and cadmium and 0.5 µg/g for lead.

The companies that provided results of metals testing reported arsenic, cadmium, lead, and mercury concentrations, as shown in Table 5 (except for mercury), which were obtained from an ISO-certified third-party lab. The state action limits used for comparison in the lab reports are also included in Table 5. The instrumentation used by the labs, ICP-MS, atomic fluorescence spectroscopy (AFS), or unknown, is shown in the table. We advise caution in comparison with our ICP-MS results from Table 2 with other results. We dried the hemp filler before analysis while the analysis of the hemp from the 4 reporting brands either note that they analyzed as received or make no note. For comparison purposes we treated all samples reported as analyzed as received. Therefore, we included information on moisture content provided in the same third-party lab reports in Table 5. We calculated moisture content in lab based on pre- and post-drying weight of the filler from 1 cigarette (*n* = 3) and included this value as well. Comparison of results should keep this difference in sample preparation in mind. Moisture content did not exceed 11% in the laboratory reports provided by 4 hemp brands.

**Table 5:** Company-provided arsenic, cadmium, and lead hemp filler results (n is unknown) [19, 20, 24, 27] compared to CDC results with the action limit used for comparison in the lab reports

Brand	Source of Results (Instrumentation)	As (µg/g)	Cd (µg/g)	Pb (µg/g)	Moisture
	**State Action Limit**	**0.200**	**0.200**	**0.500**	
**Crème Peppermint by Sugar™**	**Third party (ICP-MS)**	0.194	0.161	0.379	8.70%
	**CDC (dried; ICP-MS)**	0.124 ± 0.013	< LSTD (0.500)	0.419 ± 0.042	9.35%
**Mountain** ^®^ **Pineapple Squeeze**	**Third party (AFS)**	< LOD (0.07)	< LOD (0.07)	< LOD (0.17)	7.35%
	**CDC (dried; ICP-MS)**	0.115 ± 0.038	< LSTD (0.500)	0.297 ± 0.050	7.79%
**Redwood Reserves**	**Third party (unknown)**	< LOQ (0.010)	< LOQ (0.010)	< LOQ (0.050)	unknown
	**CDC (dried; ICP-MS)**	0.0579 ± 0.0072	< LSTD (0.500)	0.209 ± 0.017	10.0%
**Chief Stix™**	**Third party (AFS)**	ND	ND	ND	9.31%
	**CDC (dried; ICP-MS)**	0.0799 ± 0.0323	< LSTD (0.500)	< LSTD (0.200)	10.9%

arsenic (As), cadmium (Cd), lead (Pb); inductively coupled plasma-mass spectrometry (ICP-MS); atomic fluorescence spectroscopy (AFS); lowest standard (LSTD); limit of detection (LOD); limit of quantitation (LOQ); non-detect (ND)

State Action Limits shown in the table were those used in the third-party lab reports

Moisture content provided from third-party report (n is unknown) compared to our moisture content as average for 1 cig (*n* = 3)

Crème Peppermint results analyzed by ICP-MS [20] reported slightly higher concentrations for arsenic and slightly lower concentrations for lead compared to our results. Arsenic, cadmium, and lead concentrations were reported as passing by the third-party lab, although they were very close to the action limits. Mountain^®^ Pineapple Squeeze reported atomic fluorescence spectrometry results for all tested metals as < LOD [19]. We reported higher arsenic and lead concentrations that could not be explained by moisture correction alone. Redwood Reserves stated that they tested samples as received with acceptable heavy metal uncertainty acceptance limits to be within 14.5% [24]. Redwood Reserves was one of the lowest arsenic values that we reported, 0.0579 ug/g compared to their < 0.010. The lead concentration that we determined was significantly higher than the concentration reported by the contract lab. For Chief Stix™, arsenic, cadmium, and lead were reported as ND (non-detect) [27]. Our values were also lower, with arsenic being detectable and cadmium and lead < LSTD.

Differences in drying the sample can account for minor differences between our results and third-party lab report results. Some larger differences, which were all lower results from the companies’ lab reports compared to ours, would not be explained by correcting for moisture. Overall, only 4 out of 14 hemp cigarettes provided any information on metal content. All the results reported by these companies showed passing arsenic, cadmium, and lead according to state action limits. However, as discussed above, several of the other hemp cigarettes would exceed action limits from different states according to our results for arsenic and lead.

Hemp filler measurements help provide potential exposure information to consumers. However, metal chemical properties and volatility, cigarette design, filtration efficiency, and total particulate matter generated impact metal concentrations in mainstream smoke to which the user will ultimately be exposed. Further work is needed to examine the mainstream smoke from hemp cigarettes.

## Conclusions

Our previously published method for analysis of 9 metals in tobacco was successfully applied to the analysis of filler from 14 U.S. hemp cigarettes [30]. NIST RM 8210 was also analyzed and shows suitability of the method for both tobacco and hemp products as previously shown for other plant material SRMs. Except for cadmium, metals concentration ranges in hemp cigarettes filler were comparable to tobacco cigarettes and little cigars filler with some variation observed. Tukey-Kramer evaluation showed significantly lower mean concentrations from the filler of hemp cigarettes compared to tobacco cigarettes and little cigars filler for chromium, cobalt, arsenic, cadmium, and lead. The nickel mean concentration for hemp cigarettes was significantly higher compared to little cigars while there was no difference when compared to tobacco cigarettes. Uranium’s mean concentration in hemp filler was significantly higher compared to little cigars (uranium was not analyzed in the previous tobacco filler study). Beryllium and manganese had no statistical difference between hemp cigarettes and little cigar or tobacco cigarette filler. The most notable difference was hemp cigarettes filler cadmium concentrations were < LSTD while readily detectable in tobacco products filler [29, 30]. This most likely reflects the differences in uptake of cadmium between hemp and tobacco plants [41–44] but may also reflect differences in soil metals content and/or agricultural practices, including fertilization treatment during cultivation [12, 13]. When comparing to available metals data on cannabis samples in the literature, some studies’ [14–17, 47, 50] have lower results than our hemp cigarette results. Other studies have larger metal concentration ranges [45–49] compared to our hemp cigarette metal ranges. These differences can be from sampling different parts of the plant and growing location, including soil and fertilizer. There is also evidence that different hemp cultivars can affect metal uptake [41].

When hemp cigarettes arsenic, cadmium, and lead concentrations are compared to action limits set by some states, some of our results for arsenic and lead exceeded established limits. Four of the fourteen brands used in this study included third-party lab reports for arsenic, cadmium, and lead results for their hemp products. Although we report dry-mass and the companies report as-is hemp metals concentrations, our comparison showed that while many results are similar to ours, there are some third-party results that are significantly lower than our results. Such differences could result from batch-to-batch differences of the hemp product or the respective analytical methodologies.

This manuscript provides the first report of selected metals data for hemp cigarettes filler. The 14 brands represent a convenience subset of hemp cigarettes available in the United States. Reporting metal concentrations from these products using a well-validated analytical method is important for public health and consumer safety as these metals have various negative health effects such as carcinogenicity, sensitization, and inflammation. With varying filter material and design and filler mass per cigarette, the metals concentrations from the total particulate matter of the smoke from these hemp cigarettes would provide more information on human exposures. A subsequent manuscript of evaluation of metals concentrations in mainstream hemp cigarettes smoke is underway.

## Data Availability

The datasets used and/or analyzed during the current study are available from the corresponding author on reasonable request.

## Abbreviations

THC: Tetrahydrocannabinol
CBD: Cannabidiol
Be: Beryllium
Cr: Chromium
Mn: Manganese
Co: Cobalt
Ni: Nickel
As: Arsenic
Cd: Cadmium
Pb: Lead
U: Uranium
RM: Reference Material
XRF: Polarized energy dispersive x-ray fluorescence
HPHC: Harmful and Potentially Harmful Constituents
TFM: Modified polytetrafluoroethylene
PFA: Perfluoroalkoxy resin polymer
QQQ-ICP-MS: Triple quadrupole-inductively coupled plasma-mass spectrometry
QC: Quality control
LOD: Limit of detection
SRM: Standard Reference Material
ICP-OES: Inductively coupled plasma-optical emission spectroscopy
LSTD: Lowest standard
RSD: Relative standard deviation
PTP: Proficiency testing program
LRL: Lowest reportable level
FAPAS: Food Analysis Performance Assessment Scheme
HSD: Honestly significant difference
USDA: United States Department of Agriculture
USP: United States Pharmacopeia
PDE: Permitted daily exposure
AFS: Atomic fluorescence spectroscopy
ND: Non detect
LOQ: Limit of quantitation
